# Landmark-based retrieval of inflamed skin vessels enabled by 3D correlative intravital light and volume electron microscopy

**DOI:** 10.1007/s00418-022-02119-8

**Published:** 2022-06-29

**Authors:** Karina Mildner, Leonhard Breitsprecher, Silke M. Currie, Rebekka I. Stegmeyer, Malte Stasch, Stefan Volkery, Olympia Ekaterini Psathaki, Dietmar Vestweber, Dagmar Zeuschner

**Affiliations:** 1 Electron Microscopy Unit, Max Planck Institute for Molecular Biomedicine, Röntgenstr. 20, 48149 Münster, Germany; 2 iBiOs–integrated Bioimaging Facility Osnabrück, CellNanOs- Center for Cellular Nanoanalytics, University of Osnabrück, Osnabrück, Germany; 3 Department of Vascular Cell Biology, Max Planck Institute for Molecular Biomedicine, Münster, Germany; 4 BioOptic Service Unit, Max Planck Institute for Molecular Biomedicine, Münster, Germany

**Keywords:** Intravital microscopy, Dorsal skinfold chamber, Live cell imaging, Correlative light and electron microscopy, Transmission electron microscopy, Serial block-face scanning electron microscopy

## Abstract

The nanometer spatial resolution of electron microscopy imaging remains an advantage over light microscopy, but the restricted field of view that can be inspected and the inability to visualize dynamic cellular events are definitely drawbacks of standard transmission electron microscopy (TEM). Several methods have been developed to overcome these limitations, mainly by correlating the light microscopical image to the electron microscope with correlative light and electron microscopy (CLEM) techniques. Since there is more than one method to obtain the region of interest (ROI), the workflow must be adjusted according to the research question and biological material addressed. Here, we describe in detail the development of a three-dimensional CLEM workflow for mouse skin tissue exposed to an inflammation stimulus and imaged by intravital microscopy (IVM) before fixation. Our aim is to relocate a distinct vessel in the electron microscope, addressing a complex biological question: how do cells interact with each other and the surrounding environment at the ultrastructural level? Retracing the area over several preparation steps did not involve any specific automated instruments but was entirely led by anatomical and artificially introduced landmarks, including blood vessel architecture and carbon-coated grids. Successful retrieval of the ROI by electron microscopy depended on particularly high precision during sample manipulation and extensive documentation. Further modification of the TEM sample preparation protocol for mouse skin tissue even rendered the specimen suitable for serial block-face scanning electron microscopy (SBF-SEM).

**Supplementary Information:**

The online version contains supplementary material available at 10.1007/s00418-022-02119-8.

## Introduction

Tissue homeostasis is important to maintain the functionality of each organ in the body. Inflammatory events challenge this balance and often lead to drastic changes in the permeability of blood vessels, stimulating numerous rescue reactions involving neutrophils and platelets (Claesson-Welsh et al. 2021). Neutrophils leave the vessel in a controlled manner described as a transmigration process (Vestweber 2015) while platelets are sealing the endothelial barrier to prevent vascular leaks (Braun et al. 2020). Interactions of platelets and neutrophils have been observed at sites of inflammation and require further investigation (Lisman 2018) to improve understanding of the dynamics of inflammation. To this end, a mouse dorsal skinfold chamber was established, allowing high-temporal-resolution imaging with an intravital microscopy (IVM) setup (Secklehner et al. 2017). While screening the entire tissue, selective vessels with distinct behavior of transmigrating neutrophils and platelets could be observed. As the resolution of intravital microscopy is insufficient to reveal the detailed relation on a single-cell level in the region of interest (ROI), a correlative light and electron microscopy (CLEM) approach for the skin, combining IVM with subsequent ultrastructural investigation, remained to be established. This goal can only be achieved in a correlative manner, resulting in a complex workflow in which almost every step is optimized (Karreman et al. 2016). As guidance throughout the protocol, artificial and anatomical landmarks are indispensable to relocate the target vessel in the sample volume in the *x*-, *y*-, and *z* dimensions (Goudarzi et al. 2015; Luckner et al. 2018). The composition of skin tissue, a functional multilayered barrier, additionally hinders the infiltration of reagents used for fixation and embedding (Randall Wickett 2006), posing another challenge that required further optimization. The question at hand could only be answered sufficiently by three-dimensional volume electron microscopy, here using serial block-face scanning electron microscopy (SBF-SEM) (Denk and Horstmann 2004). As a consequence, extra adjustments for the sample preparation were incorporated into the workflow (Currie et al. 2022).

However, the workflow remains simple and valuable for standard transmission electron microscopy, as well as for SBF-SEM, and could be reproduced in almost any standard electron microscopy laboratory.

## Materials and methods

### Dorsal skinfold chamber IVM

All animal experiments were approved by the local authorities (LANUV, State Agency for Nature, Environment and Consumer Protection). Prior to surgery, mice were anesthetized, and the dorsal skin was shaved and chemically depilated. A titanium dorsal skinfold chamber frame (small dorsal kit, APJ trading Co Inc) was surgically attached to the disinfected skin, and a circular window area (~ 12 mm diameter) was prepared for microscopy. For further details, see Currie et al. (2022).

**Fig. 1:**
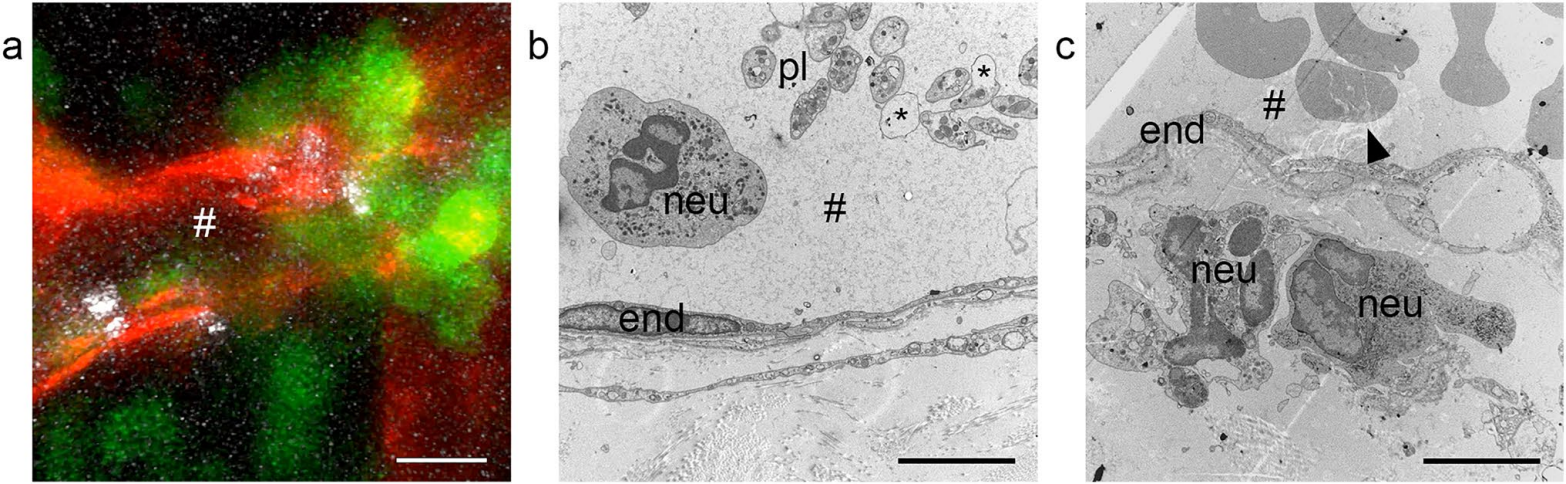
Vessel segment undergoing neutrophil extravasation during cutaneous inflammation. **a** Representative time-lapse still image of an inflamed vessel from intravital fluorescence microscopy showing the endothelial cell layer (red), transmigrating neutrophils (green), and platelets (white). Original movie in Supplementary Movie 1. **b**, **c** Electron micrographs of inflamed mouse skin sample, conventionally fixated and processed with CLEM techniques, exhibit poor preservation of the ultrastructure (asterisk marking bulging membranes of platelets) and distortion of the sections because of weak epon infiltration (arrowhead). *#* vessel lumen, *end* endothelial cell, *neu* neutrophil, *pl* platelet, *bulging membranes. Scale bar in **a** = 10 µm, scale bar in **b**, **c** = 5 µm

**Fig. 2:**
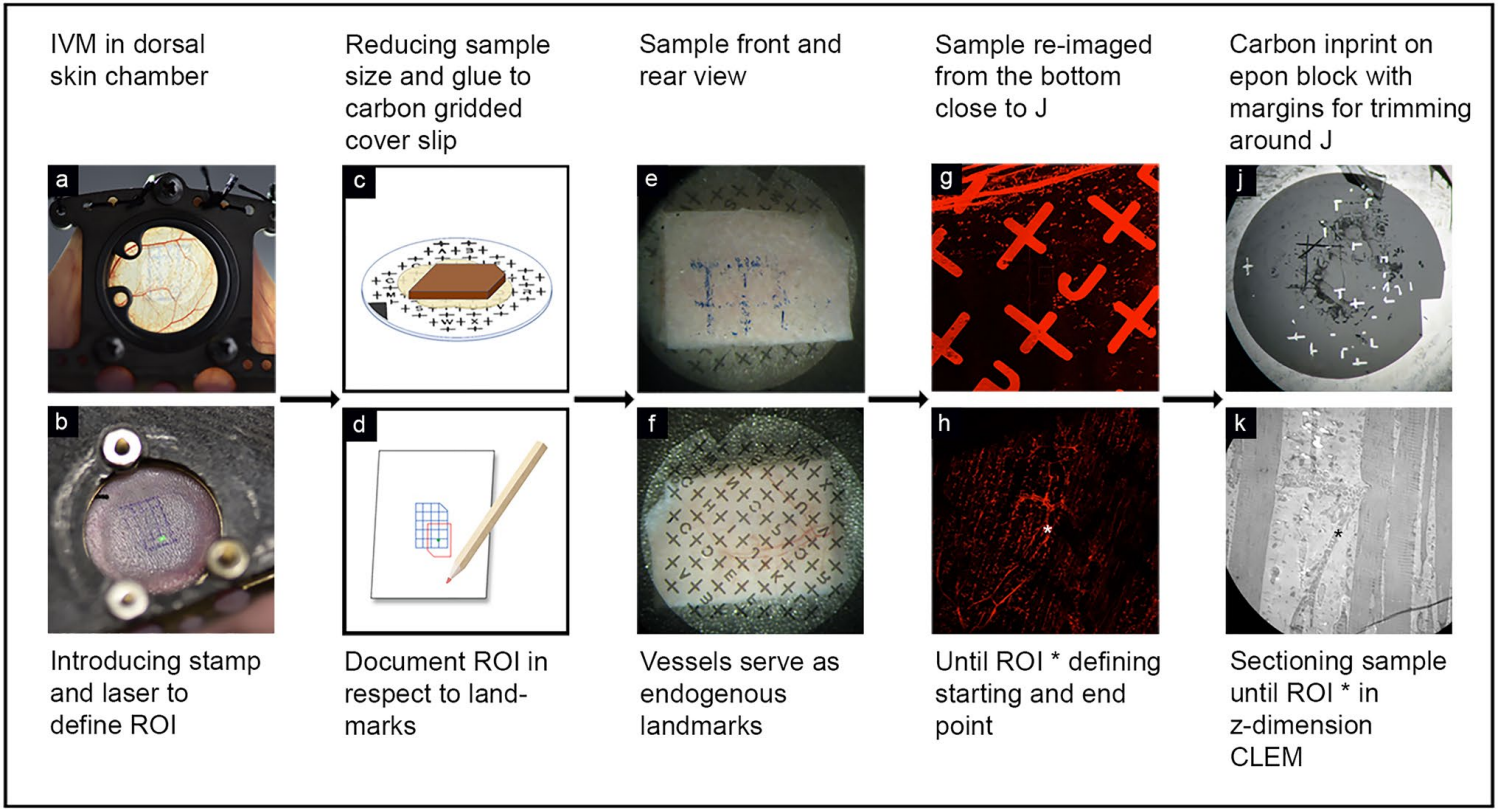
Introduction of landmarks for CLEM orientation. Illustrated workflow describing all steps introducing landmarks, which are essential to reposition ROI at the electron microscope exactly at the light microscopical location recorded by IVM in a dorsal skin chamber (**a**). The printed stamp on the skin supports to locate the laser point, which illuminates the last position of the IVM settings (ROI) **b**. After fixation, punching out, and resizing the skin to a minimum, the sample is glued to a carbon grid and marked on one corner **c**. The endogenous, visible vessels in the tissue serve as the next landmarks while repositioning the sample on the confocal stage **f**, where the fluorescently labeled vessel network in the fixed tissue serve as landmarks to relocate the ROI at the confocal microscope **g**, **h**. With the help of the carbon-gridded cover slip and its imprinted letters, the position of the ROI can be documented with respect to these very letters **d**, **e**. Together with the distance in the *z*-direction between the ROI and the coverslip, this information about the *x*–*y* location defines the starting point for the microtome sectioning **j** further illustrated in Fig. 3. The measured *z* height in the sample thus guides this approach to successfully find the correlative view between light and electron microscope (CLEM) **k**

After analgesia and 24 h of recovery, the epidermis was marked with a stamp (handcrafted), subdivided into 20 fields, thereby introducing the first artificial landmark (Fig. 2a, b). Inflammation was induced by initiating the reverse passive Arthus reaction, as described by Currie et al. (2022). Prior to anesthesia, mice were i.v. injected with 100 µL cell culture graded bovine serum albumin (75 µg BSA/g body weight) in phosphate buffered saline (PBS). Anesthetized mice subsequently received an intradermal injection of 20 µL rabbit anti-BSA (30 µg/mouse, MP Biomedicals) antibody into the stamp-labeled area of the back skin. Live cell imaging was performed using a Zeiss LSM 880 microscope with Airyscan fast. For this procedure, the anesthetized mouse carrying a dorsal skinfold chamber was mounted onto a custom-designed stage. Visualization of blood vessels and platelets was achieved by systemically injecting LysMeGFP mice (fluorescently tagged myelomonocytic cells) with Dylight antibodies against CD31 (30 μg/mouse, eBioscience #16–0311-85) and GPIbβ (2 μg/mouse, #X649, Emfret Analytics). To study the interaction of these immune cells, intravital time-lapse videos were recorded for 90–120 min (Fig. 1 a, Supplementary Movie). The confocal analysis focused on one distinctive postcapillary venule displaying strong neutrophil diapedesis and subsequent single-platelet binding to the vessel wall. At the end of the experiment, the anatomy of the monitored vessel segment (ROI) and the surrounding vascular network was well documented in microscopic *z*-stacks, using different magnification levels (*z*-stacks at 20× and 10×). To create an additional reference point, a photograph of the laser position within the frame of the introduced stamp was taken (Fig. 2b).

### Sample fixation and introduction of additional landmarks for CLEM orientation

After confocal IVM, mice were terminally anesthetized. The glass cover of the dorsal skinfold chamber was removed, and the skin was submerged into fixative for 2 h in a dark room (4% formaldehyde (FA) in PBS, prewarmed to 37 °C). All further steps were performed in an unlit room to prevent bleaching of fluorescence signals in the tissue. The dorsal skinfold chamber was separated from the body of the mouse, and the skin surface (epidermis) surrounding the stamp was carefully perforated with blood lancets and acupuncture needles to allow the penetration of fluids during further fixation and embedding. Next, the stamped area was punched out (12-mm dermatology skin punch), and trimmed to minimal rectangular size with a scalpel, while keeping the ROI centered. One corner of the four-sided sample was removed, serving as an additional landmark for better orientation of the specimen. The flattened tissue was glued onto a carbon gridded coverslip with letters for orientation (coating mask for finder grid; Leica, Austria) using a thin layer of 2% low-melting-point agarose, with the vascularized side of the skin sample directly facing the carbon layer (Fig. 2e, f). The agarose was solidified on ice, and the specimen was transferred to a 3-cm glass-bottomed dish (ibidi, Germany). Using the position of the stamp and the edge with the cutoff corner as landmarks, the ROI was relocated in the confocal microscope. The localization of the ROI with respect to the carbon letters on the gridded coverslip was documented in *z*-stacks at different magnification levels. To record special anatomic features of the surrounding tissue serving as additional landmarks, the anatomy of the retrieved ROI and the surrounding vascular network was captured in microscopic *z*-stacks, using different magnification levels (*z*-stacks at 20× and 10×). Finally, the distance in the *z*-direction between the vessel of interest and the coverslip was measured. The sample was then further fixed in 2% glutaraldehyde (GA), 2% formaldehyde, in 0.1 M cacodylate buffer (CB), pH 7.2, prewarmed to 37 °C.

### Epon embedding in beam capsule

For electron microscopy analysis, the sample was further processed with an adapted protocol that is also suitable for SBF-SEM (Deerinck et al. 2010) (Goudarzi et al. 2017), as summarized in Table 1. Contrast enhancement was specifically achieved by keeping the specimen on a warm heating plate at 40 °C during all incubation times starting with postfixation reagents (osmium tetroxide OsO_4_, thiocarbohydrazide TCH, lead aspartate; OTOTO) and followed by several washing steps. Uranyl (UA) en bloc staining was performed at 4 °C overnight, followed by dehydration in ethanol and acetone at room temperature and subsequent incubation with increasing concentrations of epon. The last epon infiltration solutions were mixed with 0.07% (w/w) Ketjenblack (KB; TAAB Laboratories Equipment Ltd) to improve the conductivity of the resin, as required for SBF-SEM (Nguyen et al. 2016). Finally, a beam capsule was attached above the ROI. After resin polymerization, the gridded glass coverslip was blown off by sequential plunges in liquid nitrogen and hot water. Ideally, the carbon imprint remained on the sample surface. See Table 1 for a more detailed description of the individual steps for the protocol.

**Table 1:** Step-by-step protocol for optimized mouse skin fixation and SBF embedding

Step no.	Solution	Time	Temperature	Comment
1	4% FA in PBS, 37 °C	2 h	RT	Prefixation in the dark to keep fluorescence
2	Cut out part with ROI, glue on coverslip with agarose, and find back ROI in fluorescence microscope with respect to the carbon grid			
3	2% GA + 2% FA in 0.1 M CB, 37 °C	1 h + overnight	RT + 4 °C	Final fixation
4	1% FA in 0.1 M CB	Until embedding	4 °C	Storage
5	Demineralized water	3 × 5 min	40 °C on hot plate^a^	In four-well plate to reduce chemicals
6	2% OsO_4_ + 1,5% KFeCN (potassium ferricyanide(III)) in demineralized water)	30 min		
7	Demineralized water	3 × 10 min		
8	1% TCH in demineralized water	15 min		Prepare the total volume you need for the day at the beginning of the embedding and let it cool down to 40 °C
9	Demineralized water	3 × 10 min		
10	1% OsO_4_ in demineralized water	30 min		Transfer sample to fresh four-well plate
11	Demineralized water	3 × 10 min		
12	1% TCH in demineralized water	20 min		
13	Demineralized water	3 × 10 min		
14	1% OsO_4_ in demineralized water	30 min		Transfer sample to fresh four-well plate
15	Demineralized water	4 × 10 min		
16	0.5% UA in 25% methanol	Overnight	4 °C	Transfer sample to fresh four-well plate
17	Demineralized water	3 × 10 min	40 °C on hot plate^a^	
18	Lead aspartate	60 min		Incubation at 60 °C is not possible because the agarose would melt
19	Demineralized water	2 × 5 min		
20	70% ethanol	Overnight	4 °C	
21	90% ethanol	2 × 15 min	RT	Without agitation, to prevent detachment of the sample
22	96% ethanol	2 × 15 min		
23	100% ethanol	3 × 30 min		
24	100% acetone	3 × 10 min		Transfer sample to glass dishes (40 mm diameter)
25	Epon–acetone mixture 1 + 3	1 h		
26	Epon–acetone mixture 1 + 1	1 h		
27	Epon + 0.7% Ketjen Black (KB)–acetone mixture 3 + 1	1 h		Mix the total volume of epon you need with KB (volatile powder!)
28	Epon, pure + 0.7% KB	1.5 h		Plus 15 min under vacuum to facilitate infiltration of resin
29	Epon, pure + 0.7% KB	Overnight		Plus 15 min under vacuum to facilitate infiltration of resin
30	Epon, pure + 0.7% KB	2 h or longer		Plus 15 min under vacuum to facilitate infiltration of resin
31	Place on top of a beam capsule (without lid and bottom)	Overnight	60 °C	
32	Fill the capsule with epon–KB to 3/4	6 h		
33	Insert a label with sample name and fill up with pure epon without KB	3 days		The label is not readable in KB

The table summarizes all the steps for fixation and embedding of murine skin. The steps were modified to counteract the loss of contrast after conventional treatment of the CLEM sample (Fig. 1b, c). Major changes include the raise of temperature of the reagents and inclusion of OTOTO counterstaining (step 5–15 and 17–19), rendering the sample suitable additionally for SBF-SEM

^a^This needs to be done carefully, because raising the temperature risks detachment of the sample, as the low-melting-point agarose has a tendency to melt at 40 °C

### Target sectioning

**Fig. 3:**
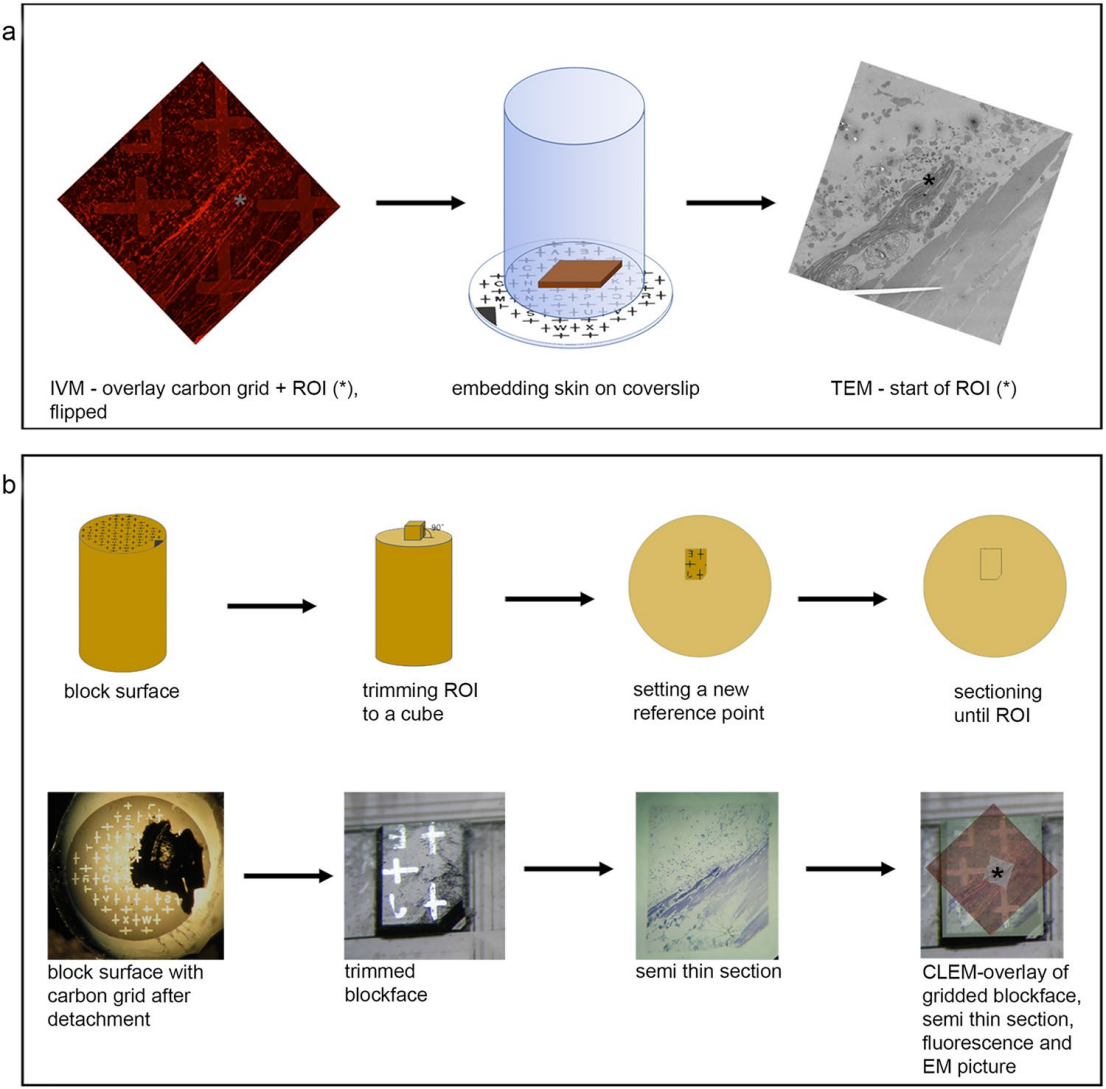
Target trimming and sectioning. **a** Outline of the CLEM challenge: retrieve a specific area from skin tissue, recorded by IVM (left panel), repositioned several times through the entire sample preparation (middle panel) and reimaging the area exactly at the ROI (*) in the electron microscope (right panel). The trimming and sectioning of the embedded sample block needs to be done in a controlled manner as further illustrated in **b**. After detachment from the coverslip, the block surface is covered with traces of the carbon grid (left). The resin material is trimmed away from the ROI, identified by the letter of the carbon imprint, resulting in a small square block (second image). One corner is cut off, defining a new reference point. Next, the ROI is approached by careful sectioning with µm/nm step size, until the *z*-height of the vessel of interest is reached. The approach is documented with 200-nm-thick sections, stained with toluidine blue, which are continuously compared with the pattern of the vessel network as imaged in the confocal microscope on the prefixed sample. The overlay of all corresponding layers (fluorescence image, carbon grid, and semithin and thin section) prove that the vessel has been retrieved in a correlative mode and allows further investigation at higher resolution in the electron microscope

The carbon imprint of letters and crosses on the block surface, now preserved as a mirror picture, is indispensable for retracing the ROI during ultrathin sectioning (Fig. 3). Therefore, all steps modifying the block need to be documented carefully (see also Fig. 3b).

First, unlike standard protocols, the block was trimmed to a cubic and not pyramidal shape, so that the dimension of the sections will not increase with further cutting into the specimen. Using a diamond knife (Ultratrim 90, Diatome, Switzerland), the size of the specimen was reduced to maximal 1 × 1 mm^2^ in the *x*–*y* direction. One corner of the block was removed, creating a new reference point, as the carbon mask disappears with the first sections. The forward trimming and sectioning steps were all documented. At levels of interest, sections of 200 nm thickness were collected and stained with toluidine blue. Samples were inspected with a light microscope to identify structures within the vascular network that match the anatomical landmarks of the vasculature recorded by confocal fluorescence microscopy (*z* stacks). In the ROI, the overlay of all images (carbon grid, fluorescence image, light microscopy and electron microscopy image) verified that the target region was approached successfully.

### Transmission electron microscopy

**Fig. 4:**
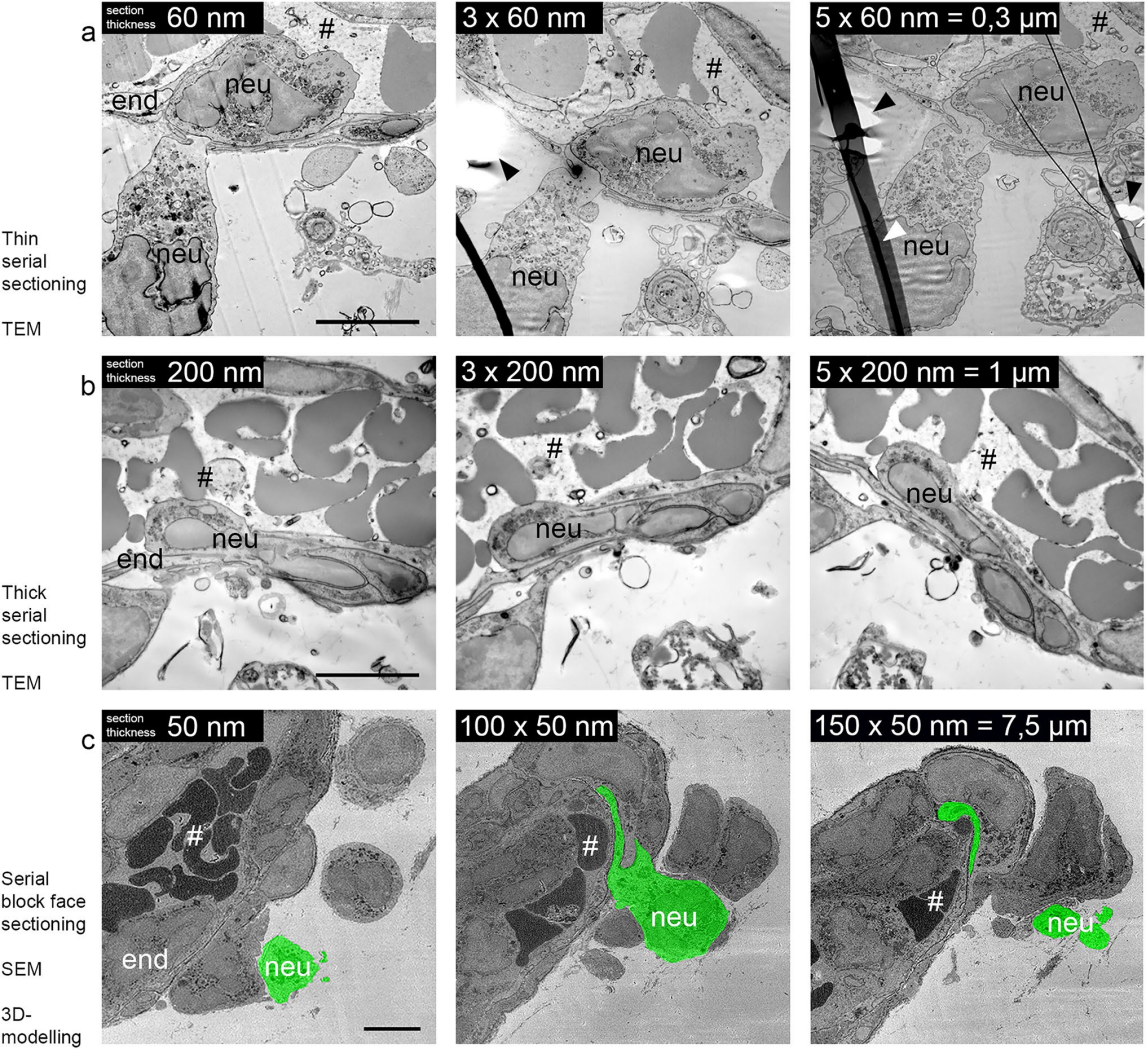
Analyzing the ROI by thin sectioning, thick sectioning, or serial block-face sectioning. Electron micrographs of successfully recovered CLEM vessels of inflamed mouse skin. Throughout the vessel, several cellular interactions can be observed and studied in more detail with respect to the surrounding tissue components such as the endothelial cell (end) and transmigrating neutrophil (neu) leaving the lumen of the vessel (#). To provide more “volume” information, several techniques can be applied. Serial consecutive sections (60 nm thin or 200 nm thick) can be used to follow structures in 3D on a normal transmission electron microscope **a**, **b**. Physical distortions occur regularly, marked by arrowheads. An alternative approach is automated sectioning by SBF-SEM. The sample block is continuously cut via an ultramicrotome, accommodated within the instrument. The scanning detector scans the surface after every section, resulting in a large 3D volume of the selected ROI that can be recorded and analyzed as single sections **c**. Here, the contour of a transmigrating neutrophil is highlighted in green, and additionally annotated in Supplementary Movie 3. Scale bar = 5 µm

Thin sections of 60 nm of the ROI were collected on 1 slot-filmed copper grids, counterstained with lead, and imaged in the electron microscope (Tecnai 12-biotwin, Thermo Fisher Scientific Inc.). No counterstaining was needed if the samples were processed according to the OTOTO protocol. Additionally, consecutive serial sections of 60 nm or 200 nm thickness were collected and analyzed to provide more preliminary “volume” information in 3D (Fig. 4a, b). Representative images were taken with a 2k charge-coupled device (CCD) camera (Veleta, EMSIS, Münster, Germany) and arranged with Adobe Photoshop without further processing.

### Serial block-face scanning electron microscopy

Preselected correlative samples were trimmed to a size of approximately 500 × 500 × 500 µm^3^ and mounted on an aluminum specimen holder using a two-component silver conductive epoxy adhesive in a ratio of 1.25:1 (Wanner et al. 2016). The samples were coated with a 30-nm thin layer of gold in a sputter coater (ACE EM 600, Leica, Germany). The trimmed sample was mounted in the sample holder of the SEM (JSM-7200F, JEOL, Japan) integrated ultramicrotome stage (3View2XP, Gatan, USA). To minimize the imaging time, the trimmed sample block was aligned such that the course of the blood vessel was as parallel to the knife edge as possible, based on the previous TEM analyses (Fig. 2). An additional helpful tool in aligning the sample block was the reference landmark (cut-off edge on one side of the square sample block).

Optimizing imaging parameters for SBF-SEM is highly dependent on the type of sample. To achieve the best contrast and resolution possible, both high accelerating voltages and high vacuum are desirable. However, this approach could increase the possible charge accumulation in regions of unstained and nonconductive biological tissue or empty resin areas. Whereas backscattered electrons are detected for the final image acquisition, secondary electrons are more prone to charging artifacts (Kim et al. 2008). Utilizing the secondary-electron detector unit within the microscope, charge accumulation manifested as intense brightness can therefore be detected more easily and precautions be taken. The sample investigated here proved to be stable under imaging parameters of 1.5 kV accelerating voltage, utilizing a 30 nm condenser aperture, HV conditions of 10 Pa, and a positive bias of 400 V. The pixel resolution of each ROI was set to 5 nm, in between ablation of 50-nm slices with a dwell time of 2 µs. The multi-ROI acquisition was controlled by Gatan Digital Micrograph software (version 3.32.2403.0). The same was used to convert the raw data (.dm4) into 16-bit TIFF files using a custom script command. Further processing of the datasets, including alignment, rendition, and extraction of subvolumes and segmentation, was handled using Microscopy Image Browser (version 2.7) (Belevich et al. 2016). To keep the considerable data size to a minimum, all datasets were converted to 8-bit TIFFs and binned to a factor of 2 or 4. In case high resolution was required, an unbinned subvolume of the ROI was isolated from the raw dataset. To keep imaging times short, the dwell times during acquisition can be reduced at the higher noise ratios. The noise can be removed via edge-preserving filters, e.g., anisotropic diffusion. Subsequent graylevel image histogram normalization can restore sufficient contrast for semiautomatic segmentation tools (e.g., watershed or global threshold) based on specific gray values. The final models and image stacks were exported for volumetric visualization in Amira 3D (version 2021.1, Thermo Fisher Scientific Inc.).

## Results and discussion

IVM of inflamed skin tissue allows live cell imaging over a long time period (Fig. 1a, Supplementary Movie 1). In response to the inflammation stimulus, neutrophils leave vessels, mainly through partial openings of endothelial junctions (paracellular transmigration route). Only in distinct vessels could increased numbers of platelets, obviously attracted by the transmigration process of neutrophils, be observed. As this co-interaction is not a general phenotype, imaging of the same position seen in IVM by electron microscopy at higher resolution was required. This observation cannot be captured in an unsystematic sectioning process, so a CLEM approach is the only way to retrace the same event (Kopek et al. 2017). The first attempts allowed us to relocate the vessel of interest, similarly to what we described earlier for zebrafish tissue (Goudarzi et al. 2015). However, we encountered unsatisfactory preservation, contrast, and physical distortions of the sample (Fig. 1b, c). Two major entry sides of the CLEM tissue block were masked, viz. the epidermis and the glued side on the cover slip, where fluids can hardly penetrate (Fig. 2 c, Supplementary Fig. 2). At these sides, infiltration of fixatives and contrasting reagents were abolished, resulting in a dramatic lack of conservation and contrast compared with previous samples (Frye et al. 2015). It was not possible to further minimize the sample dimension, for example, by vibratome sectioning, as the course of the vessel could not be accurately located into a certain volume (Sousa et al. 2021). Therefore, we started to optimize the protocol step-by-step (Table 1), mainly inspired by the staining methods used for SBF-SEM (Denk and Horstmann 2004).

To overcome the infiltration barrier of the fixed skin, we opened the epidermis by introducing small lesions with acupuncture needles and blood lancets. A major improvement was achieved by increasing the temperature during sample preparation, which led to better infiltration of reagents, including osmium tetroxide, as exemplified in Supplementary Fig. 2. The risk that raising the temperature could alter the reactivity of the chemicals was known, but a compromise between altered reactivity and the need for the fixatives to reach the restricted regions had to be found. The morphological appearance, obtained with the adjusted protocol, showed no sign of proteolysis compared with smaller sized, conventionally processed control skin samples (not shown here). It should be mentioned that raising the temperature additionally risks detachment of the sample, as the low-melting-point agarose has a tendency to liquify at 40 °C.

Artificially introduced landmarks, such as the stamp on the skin, removal of corners from the sample, and carbon gridded coverslip, were indispensable for retracing the fluorescently labeled ROI from the IVM sample in the electron microscope sample. The imprint of the carbon cover slip was not always retained perfectly on the block surface, but most of the times the remnants were sufficient to decide on the trimming area (Figs. 2j, 3b). Trimming with a 90° diamond knife was essential, resulting in a minimized square size of the block, which does not change shape while sectioning deeper into the tissue. Every step was photographed and contributed to rebuilding missing information.

The fluorescently labeled vascular network of the sample turned out to be a valuable guide as well (Fig. 2h), especially for retracing the ROI under the confocal microscope in the mildly fixed and immobilized skin sample. Moreover, the anatomy of the network facilitated localization of the vessel of interest during targeted sectioning while comparing 200-nm sections stained for light microscopy with fluorescent images in the confocal stack (Fig. 3).

A stepwise sectioning approach is recommended as the *z*-dimension of the fluorescence data cannot be transferred 1:1 to the EM specimen, since the applied treatments (fixation, heating, dehydration, embedding, etc.) alter the tissue proportions. The sample dimension changes to an unpredictable extent, especially as skin is a multilayered tissue with varying tissue composition in each layer, so each layer reacts differently to the treatment. Even swelling effects beyond those normally expected shrinkage  were observed.

Taken together, there is no (and probably never will be a) standard CLEM protocol. The unique characteristics of the specimen define the adjustments required to optimize the protocol. We performed ten experiments to establish the presented protocol and successfully reproduced the approach eight times.

Depending on the question, ultrathin serial sections might already answer the original research question. To generate volume data (in 3D), serial sectioning can be quite unpleasant when the section quality is disturbed by folds and holes (arrowheads in Fig. 4), sections get lost during retrieval, or the selected area does not develop throughout the series of images. As a consequence, the *z*-dimension that can be examined is quite limited. For more complex questions, the sample can be further analyzed by SBF-SEM, resulting in a 3D volume data stack that can be inspected and annotated similar to the confocal volume (Fig. 4, Supplementary Movie 3). In particular, samples with many extracellular spaces, e.g., blood vessels, or less heavily stained subcellular regions are increasingly prone to higher electron doses. To mitigate possible charging artifacts during image acquisition, it is therefore necessary to introduce conductive fillers in the epon resin and keep the electron dose per unit area to a minimum while maximizing resolution. In the absence of variable pressure modes (Griffin 2007) and focal charge compensation (Deerinck et al. 2018), it is possible to bias the sample stage with a positive charge to account for the negatively charged electron accumulation (Reimer 2019). Prior to the final SBF-SEM acquisition, the imaging parameters must be optimized as beam-induced artifacts can only be fully evaluated after a few imaging cycles.

The image size was highly dependent on the course of the blood vessel, thus fluctuating throughout the overall data acquisition. The natural irregular course of the vessel, due to the cutting plane of the diamond knife, occasionally led to multiple regions in which the vessel surface was exposed before ultimately fusing. To prevent loss of information due to the inherently destructive slicing of SBF-SEM, several ROIs along the vessel were imaged and adjusted frequently to the morphology of the vessel. This process kept the imaging time to a minimum. The overlap between the different selected tiles was kept as small as possible to prevent large areas from being exposed twice to the electron dose. Overall, 1200 consecutive slices (60 µm) were acquired, on numerous occasions with up to three different ROIs being imaged. Very useful in this context was remote control of the SBF run, which allows readjustment of the actual scanning area with respect to vessel progression into the sectioned block volume.

The dataset shown in Fig. 4 and Supplementary Movie 3 represents a subset of 153 slices and covers a volume of 68 × 53 × 7.6 µm^3^ (the corresponding original IVM data are published in Currie et al. 2022, Fig. 1, and Supplementary Movie 1).

## Outlook and Conclusions

Will CLEM become a routine approach comparable to confocal microscopy, accessible to all scientists? This was anticipated by Karreman (2016). Small, service electron microscopy units are still not routinely preparing CLEM samples, and when this is requested by customers, new challenges with changing model systems, changing research questions, and collaborating partners arise. Therefore, we tried to establish a simple workflow based on our own experience and with regard to the actual research question, trying to implement published tools (Muller-Reichert and Verkade, 2021).

The workflow had be optimized carefully using a step-by-step approach and by trial and error. Despite all the problems encountered during the establishment of this workflow, the end result was reproducible and of high quality.

Recent progress in the implementation of X-ray micro-CT pre-measurements of the sample block allowed the development of a topological 3D volume well correlated to the light microscopical dataset. From this dataset, even the sectioning angle and depth can be derived and programmed to a motorized microtome (https://github.com/K-Meech/crosshair).

This is probably an improvement that will make CLEM techniques more popular, as it facilitates retracing of the ROI and omits the stepwise approach. However, this requires financial investment, as not every laboratory has access to X-ray micro-CT.

SBF-SEM allows the visualization of almost unlimited 3D volumes, capturing complex cell–cell interactions. This turned out to be the fundamental instrument to solve the original biological question, as platelets could be detected in full size transferring the basement membrane barrier of the activated vessel (as documented in detail in Currie et al. 2022).

It can be expected that electron microscopy and CLEM techniques will become indispensable to study aspects of vascular dynamics in the future (Tomaiuolo et al. 2020).

## Supplementary Information

Below is the link to the electronic supplementary material.

Supplementary file 1: Supplementary Movie S1. Neutrophil extravasation during cutaneous inflammation. Confocal real-time IVM movie of neutrophil diapedesis in the dorsal skinfold chamber, as illustrated in Fig. 1a. The vessel was recorded for 1 h at a frame rate of 6 fps. Endothelial cells in red, transmigrating neutrophils labeled green, and activated platelets in white. Scale bar: 10 µm (M4V 320613 KB)

Supplementary file 2: Supplementary Fig. S2. Optimization for CLEM skin sample post-fixation, corresponding to Fig. 2, imaged at the binocular microscope. (a) after standard osmium tetroxide incubation (1% osmium tetroxide in 0.1M cacodylate buffer for 1 h at room temperature), only insufficient penetration of the dark stained reaction product into the specimen was achieved. Merely the marginal areas of the sample were penetrated by the post-fixative. (b) After additional incubation of the same sample in 2% osmium tetroxide for 1 h on a 40 °C warm heating plate, the stain infiltrated the entire tissue. (TIF 7542 KB)

Supplementary file 3: Supplementary Movie S3. Overview movie of SBF-SEM imaging of a CLEM-selected blood vessel segment. Video sequence was obtained by compilation of SBF-SEM data. The movie covers a *z*-stack height of 8 µm and shows the CLEM-relocated ROI of a selected activated vessel (the corresponding original IVM data are shown in Currie et al., 2022, 1A and Supplementary Movie 1). Details of the acquired ultrastructural volume can be inspected under different orthogonal angles. Neutrophils transmigrating over the endothelial barrier can be traced and imaged completely during the process as exemplified on one neutrophile segmented in green (also marked in Fig. 4c), here shown entirely sculptured in *z*-direction and annotated in a 3D model utilizing Amira software. (MP4 71886 KB)
