# Supplementary figures and images for: Landmark-based retrieval of inflamed skin vessels enabled by 3D correlative intravital light and volume electron microscopy
Source: Histochem Cell Biol. 2022 Jun 29;158(2):127–36. doi: 10.1007/s00418-022-02119-8 (PMC9338004; doi:10.1007/s00418-022-02119-8)

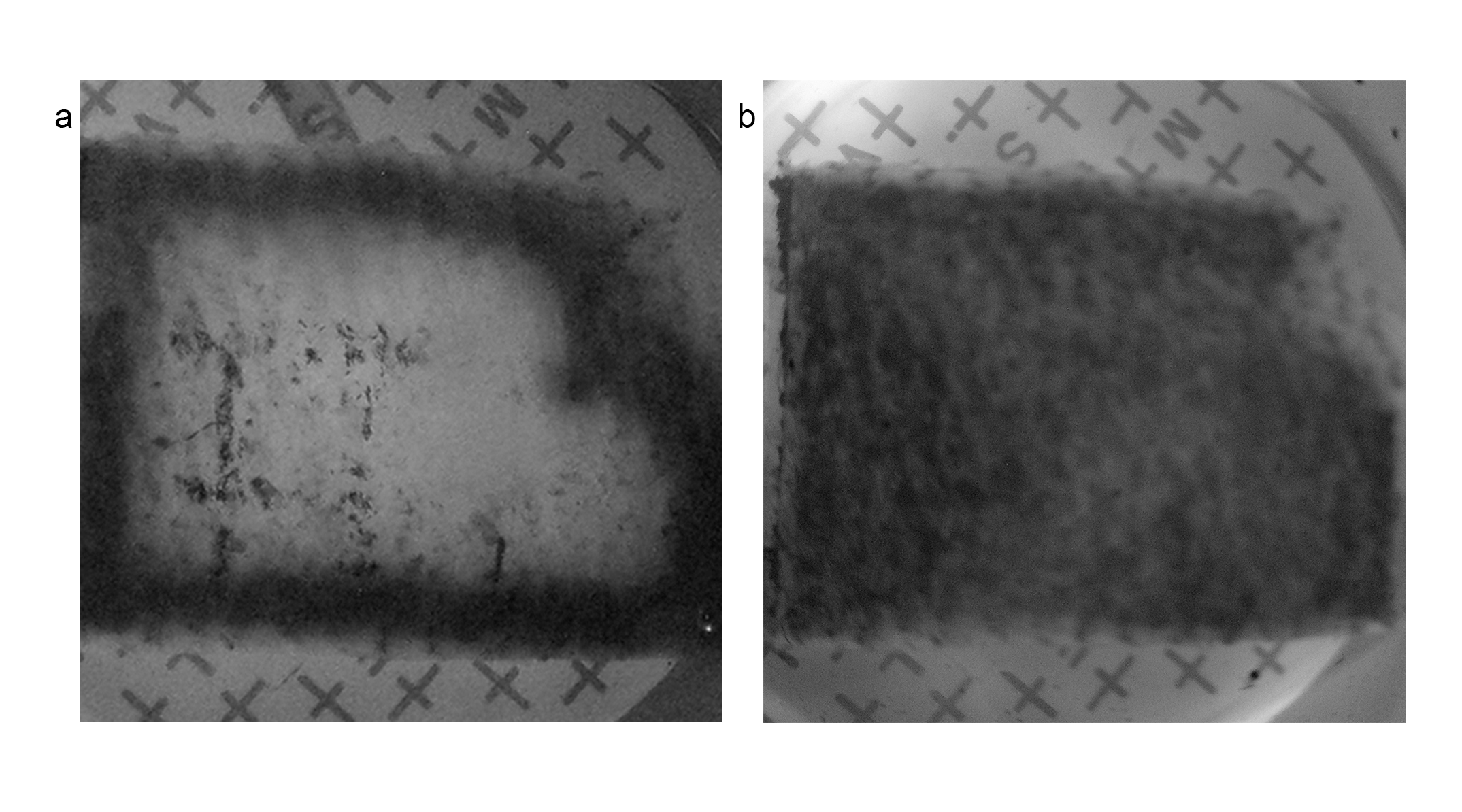

Supplement: Supplementary file 2 — Supplementary Fig. S2. Optimization for CLEM skin sample post-fixation, corresponding to Fig. 2, imaged at the binocular microscope. (a) after standard osmium tetroxide incubation (1% osmium tetroxide in 0.1M cacodylate buffer for 1 h at room temperature), only insufficient penetration of the dark stained reaction product into the specimen was achieved. Merely the marginal areas of the sample were penetrated by the post-fixative. (b) After additional incubation of the same sample in 2% osmium tetroxide for 1 h on a 40 °C warm heating plate, the stain infiltrated the entire tissue. (TIF 7542 KB) [file 418_2022_2119_MOESM2_ESM.tif]
